# Jottings from Sweden and Denmark

**Published:** 1890-05-17

**Authors:** 


### JOTTINGS FROM SWEDEN AND DENMARK.

A HOME for convalescent women is to be erected at Fredensborg, Denmark, Mr. J. Hegel, bookseller and publisher, Copenhagen, having liberally given 250,000 kr. for this purpose. The home, which is to be an extension of a similar establishment for some time past carried on at his country house by Mr. Hegel, will be located in a villa with good grounds, purchased with this view. The new home will be under the superintendence of Professor Studsgaard, from the Copenhagen Kommune Hospital, who will act in conjunction with another doctor selected by him from the same hospital, and it is the intention that two doctors from this hospital shall always form the directors of the home. The home will be used through the whole of the year, and will have good accommodation for ten patients at a time. The home for convalescent women will not absorb the whole of Mr. Hegel's handsome donation, and it is intended to allow the balance to accumulate in order to be able to erect a similar home for male convalescents by-and-bye.

The town of Helsingfors, Finland, boasts an excellent institution in its Working Home for Children. From eight o'clock in the morning children from the national schools and from poor homes are received at the home and employment provided for them. The younger ones are engaged in making boot mats, &c., and the older ones in sewing, shoemaking, and tailoring. The children are always busily engaged at the long tables in the roomy and lofty room, under the superintendence of the matron of the home and a couple of governesses. The children are also instructed in singing, and several times a week in tailoring and shoemaking. The children not only learn to repair their own clothes and boots, but also to make new ones. As remuneration for their work, the children obtain good strengthening food three times a day. The meals are served in a special room, and the children help the cook, both in cooking and in laying the table, &c. The institution is supported by voluntary contributions.

The Gamle Bakkehus Asylum for Idiots at Copenhagen has recently issued its report for 1888-89 for a period of fifteen months. During that period 14 inmates had either died or left the asylum, the total number of inmates being 135. One of those pupils who had left the institution showed no signs of getting on, either theoretically or practically, after the lapse of two years, so the parish withdrew their grant, and she had to return to her home. With regard to the newcomers, the report contains some interesting particulars. The youngest was seven and the oldest twenty-four years old. Six could not speak at all, three could pronounce a few single words, and four could say a sentence. Of seventeen, only four were able to carry on a fairly coherent conversation. Imbecility had, as a rule, existed from birth, but with a few it had appeared between the first and the fourth year, generally caused by fits, in one case by inflammation of the brain, and epilepsy was partly connected with the imbecility of several cases. In eight cases affections of the central nervous system could be traced in the nearest relatives of the idiots; only in one case the parents were related (first cousins). With several the defect of brain power was due to bodily abnormities, *e.g.*, fingers or toes being grown together, ettsophtalmy on both eyes, deformities of the lower jaw, abnormally bent knees, &c.

Regular and continuous reports are now being kept of all the pupils, and interesting materials as to the gradual development of a number of imbeciles are expected to result from the half-yearly notes made by the teachers and the manager about the pupils' general state, development and progress in the separate branches. A collection of photographs of the inmates has also been started. The report also gives full particulars about the working in the different sections, and the system which is adopted. In the experimental class, the instruction comprises practice with reference to shape, colour, qualities, substances, objects, pictures, picture reading, articulation, slöjd and gymnastics. From this class the pupils may be advanced to the preparatory class, where the pupils are instructed in the first rudiments of spelling, writing, arithmetic and religion. The instruction in dancing is also an item of some importance, more probably than most may be inclined to think, as it helps to give the imbeciles a certain amount of lightness of carriage of which they, as a rule, are entirely devoid. In the theoretical class geography is added, the pupils beginning by trying to become acquainted with larger and larger areas by personal presence, the same practice being afterwards repeated in the same order on large and special maps. Orthography is also added. In the practical class such pupils are instructed as do not seem suitable for the theoretical class. The work done by the pupils is of the most different kind, comprising clothes-pegs, boarding, scarves, lace, etc., etc.

The Sabbatsberg Infirmary at Stockholm, has recently issued its report for the year 1888. Statistics are generally somewhat behind time in that part of the world. The infirmary has 340 beds, and the number of patients had been 3,305, of whom 2,597 had left the hospital cured; 165 without having been restored to health; and 278 had died. The expenses were 153,550 kr. (1,814 equal to £1), of which repairs, transport of sick persons, and funerals, &c., account for 11,122 kr., leaving a sum of 142,428 kr. for the regular working of the infirmary. This expenditure gives, with 100,297 sick days, an expense per patient per diem of 148 kr. (or about 1s. 7d. per day). The daily cost of food per patient was 0,373 kr. (or about 5d.), and the cost of medicine was 0,087 kr. (or rather more than 1d. per patient per day).

A movement has for some time been on foot in Stockholm to get a separate children's hospital in the Swedish capital. Sufficient funds have now been collected to make a commencement, although on a very modest scale. A house has been taken for five years, and is now being fitted up in the hope that the little hospital may begin its work as speedily as possible. Dr. S. von Hafsten has promised to officiate free of charge for five years as doctor to the hospital, and the apothecaries supply free medicine. A large engineering firm has presented the hospital with the necessary beds, and many other proofs of interest and sympathy have been given.

The Swedish mechanical gymnastic system has just completed the first twenty-five years of its existence, Dr. J. G. W. Zander having founded his institute at Stockholm at the beginning of 1865. Considerable time elapsed, however, before it attained its present dimensions and reputation, and before a larger number of doctors gained confidence in its advantages; and it is only during the last ten years that the system has been adopted abroad. Besides the many Swedish institutes, there are now such erected, on Dr. Zander's principle, in London, Christiania, Copenhagen, Abo, Helsingfors, St. Petersburg, Moscow, Hamburg, Berlin, Breslau, Dresden, Wurzburg, Frankfort, Carlsruhe, Baden-Baden, Plorgheim, Mannheim, Vienna, Buda Pest, Baltimore, and Buenos Ayres, to which may be shortly added Wiesbaden, Munich, and New York.

